# Sitting Behavior and Obesity: Evidence from the Whitehall II Study

**DOI:** 10.1016/j.amepre.2012.10.009

**Published:** 2013-02

**Authors:** Richard M. Pulsford, Emmanuel Stamatakis, Annie R. Britton, Eric J. Brunner, Melvyn M. Hillsdon

**Affiliations:** a Sport and Health Sciences, College of Life and Environmental Sciences, University of Exeter, Exeter, Devon, United Kingdom; b Department of Epidemiology and Public Health, University College London, London, United Kingdom; c Physical Activity Research Group, Population Health Domain, University College London, London, United Kingdom

## Abstract

**Background:**

Prospective studies report associations between indicators of time spent sitting and obesity risk. Most studies use a single indicator of sedentary behavior and are unable to clearly identify whether sedentary behavior is a cause or a consequence of obesity.

**Purpose:**

To investigate cross-sectional and prospective associations between multiple sitting time indicators and obesity and examine the possibility of reverse causality.

**Methods:**

Using data from the Whitehall II cohort, multiple logistic models were fitted to examine associations between prevalent obesity (BMI ≥30) at Phase 5 (1997–1999), and incident obesity between Phases 5 and 7 (2003–2004) across four levels of five sitting exposures (work sitting, TV viewing, non-TV leisure-time sitting, leisure-time sitting, and total sitting). Using obesity data from three prior phases (1985–1988, 1991–1993; and recalled weight at age 25 years), linear regression models were fitted to examine the association between prior obesity and sitting time at Phase 5. Analyses were conducted in 2012.

**Results:**

None of the sitting exposures were associated with obesity either cross-sectionally or prospectively. Obesity at one previous measurement phase was associated with a 2.43-hour/week (95% CI=0.07, 4.78) increase in TV viewing; obesity at three previous phases was associated with a 7.42-hour/week (95% CI=2.7, 12.46) increase in TV-viewing hours/week at Phase 5.

**Conclusions:**

Sitting time was not associated with obesity cross-sectionally or prospectively. Prior obesity was prospectively associated with time spent watching TV per week but not other types of sitting.

## Introduction

Obesity is an established risk factor for several major chronic conditions, including cardiovascular and metabolic outcomes and certain cancers.^1^ Moderate- to vigorous-intensity physical activity (MVPA) has an established protective effect against a range of such outcomes and associated risk factors, including obesity.^2,3^ An emerging body of evidence suggests that sitting may be linked to cardiometabolic risk independently of MVPA.^4,5^

Prospective studies have demonstrated positive associations between indicators of sitting behavior and mortality,^6–9^ cardiovascular disease,^10,11^ and metabolic disease including type 2 diabetes,^12–15^ which are independent of MVPA. Cross-sectional studies have reported consistent associations between sitting behavior and obesity prevalence,^16,17^ whereas some prospective studies have reported sitting to predict incident obesity or positive changes in bodyweight or adiposity.^14,18–20^ Nevertheless, evidence for associations between sitting and cardiometabolic risk is equivocal: other studies have shown that body weight status can predict sitting time,^21^ sedentary lifestyle,^22^ and reduced levels of physical activity,^23,24^ but sitting may not predict future obesity.^21^

A recent longitudinal study^25^ looking at the associations between TV viewing in early adulthood and cardiometabolic risk profiles in middle age also found that once the analyses were adjusted for baseline BMI, there was little evidence of an association. BMI and waist circumference have been found to explain most of the association between time spent sitting and cardiometabolic risk factors.^26^ Various types of sitting that vary in duration and pattern may have differential associations with health outcomes.^27^ Despite this, only one previous prospective study^14^ has separately examined whether different types of sitting are differentially associated with obesity. Other studies have examined all sitting behaviors combined or a single type of sitting, most commonly TV viewing and/or recreational screen time. Further, no studies have examined the prospective associations between obesity and various types of sitting.

The aim of the present study was to add to the current literature by examining various types of sitting and the direction of any relationship with obesity. Drawing on data from two measurement phases of the Whitehall II cohort study, the cross-sectional and prospective associations of five sitting exposures with obesity were examined. In addition, data from earlier measurement phases were used to examine the hypothesis that obesity may determine various types of sitting behavior rather than sitting behavior determining obesity.

## Methods

### Participants and Study Background

The Whitehall II study was established in 1985 to address the specific biological mechanisms that account for observed social inequalities in cardiovascular disease and diabetes.^28^ The sample included 10,308 people (6895 men and 3413 women) from clerical and office support grades, middle-ranking executive grades, and senior administrative grades. All participants provided written informed consent, and the University College London research ethics committee approved the study.

Baseline examination (Phase 1: 1985–1988) involved a questionnaire and a clinical examination, and subsequent measurement phases have alternated between a mailed questionnaire alone and a mailed questionnaire accompanied by a clinical examination. Detailed measures of physical activity and sitting behavior were undertaken during Phase 5 of data collection between 1997 and 1999 (*n*=7830); height and weight at baseline (1985–1988); and Phases 3 (1991–1993), 5, and 7 (2003–2004).

### Measurement of Sitting Behaviors and Obesity

The questionnaire included items related to both occupational and leisure-time sitting behaviors. Participants were asked: *On average how many hours per week do you spend: sitting at work, driving or commuting?* and *sitting at home, e.g., watching TV, sewing, at a desk?* and responded by selecting one of eight time categories (none, 1 hour, 2–5, 6–10, 11–20, 21–30, 31–40, >40 hours). For sitting at home, participants were given an open text response option to specify two types of sitting and then selected a time category for each. Using the midpoint of each time category, five indicators of sitting expressed as hours per week were computed: (1) work-related sitting time; (2) TV-viewing time; (3) non-TV leisure sitting time; (4) total leisure-time sitting (sum of 2 and 3 above); and (5) total sitting time (sum of 1–3 above). These items have been used previously,^14^ and their validity is described elsewhere.^29^

Height (in meters) and weight (in kilograms) were measured at the clinical examinations. Obesity was defined as having a BMI, defined by standard formula, of ≥30 and was recorded at baseline and at Phases 3, 5, and 7. At baseline, participants were asked to recall their weight at age 25 years, which was used with height at baseline to estimate BMI and obesity status at age 25 years.

### Covariates

Sociodemographic covariates included age, gender, and employment grade. Employment grade in the Whitehall II study is a comprehensive marker of socioeconomic circumstance related to salary, level of responsibility, and social status.^30^ Health behaviors included smoking status (current, previous, or never a smoker); alcohol consumption (units per week); and self-rated health (excellent, very good, good, fair, or poor).

Perceived physical functioning was assessed using the SF-36^®^ and scored with the Medical Outcomes Study scoring system.^31^ The scale requires participants to consider the extent to which their health limits their ability to perform ten physical activities ranging from vigorous-intensity sporting activities to light-intensity day-to-day tasks using the responses *a lot*, *a little*, and *not at all*. These scores are summed and transformed to scale from 0 (limited a lot in performing all ten types of physical activities) to 100 (performs all ten types of physical activities without limitation). This scale has high internal consistency.^32^

Physical activity covariates included daily walking time (minutes/day); time spent in light-intensity activity and moderate- to vigorous-intensity physical activity (MVPA) in hours/week. The questionnaire asked about occupational, domestic, and leisure-time physical activities. Twenty items assessed time spent engaged in walking, cycling, stair-climbing, sports and games, domestic activity including gardening, housework, and do-it-yourself projects. Participants reported the number of occasions and total number of hours spent engaged in each activity over the previous 4-week period.

Each activity was then assigned an energy expenditure value using a compendium of physical activity energy costs.^33^ Physical activities were classified by METs, with moderate-intensity activities (e.g., heavy gardening, heavy household maintenance activities, some sports) ranging from 3 to 5.9 METs, and vigorous-intensity activities (e.g., sports) at 6 or more METs. As the energy cost of walking is dependent on walking pace and could not be determined from the Phase-5 questionnaire, walking time did not contribute to either the MVPA or leisure-time physical activity variables. Therefore, leisure-time physical activity included all other activities up to 3 METs (light housework and chores).

### Data Analyses

Because of low numbers in some of the eight categories of sitting time, the categories were collapsed into four of near equal numbers as the data permitted. Exact quartiles were not possible because of non-normal distributions. Participants were classified as obese (1) or not (0) depending on their BMI for each phase.

Separate multiple logistic regression models were fitted to examine the cross-sectional associations between each of the five sitting exposures and obesity at Phase 5. ORs and 95% CIs were estimated for each category of sitting time, by type, with the lowest group serving as the reference category. Cross-sectional analyses were limited to those who had completed both the survey and clinical examination, those who were still working in the civil service or elsewhere, and those who had not suffered any form of heart disease prior to the survey/examination. Analysis of incident obesity between Phases 5 and 7 was restricted to the same sample as cross-sectional analyses, but in addition participants who were obese at Phase 5 were excluded.

To investigate the effect of antecedent obesity on sitting behavior at Phase 5, participants were characterized as obese/non-obese at baseline, Phase 3, and at age 25 years. The sum of values from these three variables indicated the number of occasions an individual was obese prior to the baseline of the longitudinal analysis (Phase 5). Ordinary least-squares linear regression models were fitted to examine the association between occasions of obesity prior to Phase 5 (a categoric exposure variable with scores 0–3) and time spent in each of the five types of sitting at Phase 5 (as the outcomes).

Models were first adjusted for age and gender (Model A) and then further adjusted for employment grade, smoking status, weekly alcohol intake, self-rated health, physical functioning, daily walking time, and MVPA (Model B). The leisure-time physical activity variable was not included in the final models as it did not improve model fit. To test for linear trends in individual parameters, the Wald chi-square test was used, and the likelihood-ratio chi-square test was used for nonlinear relationships. Analyses were conducted in 2012 using Stata, version 11.2.

## Results

### Sample Characteristics

Participant characteristics are shown in Table 1. Logistic regression analyses showed that participants who provided complete data for the Phase-5 measurement only did not differ in baseline characteristics compared to those who provided complete data for both Phases 5 and 7.

### Cross-Sectional and Prospective Analyses

No cross-sectional associations between various sitting indicators and prevalent obesity were observed (Table 2). Between Phases 5 and 7, a total of 98 new cases of obesity were recorded. None of the five sitting exposures were associated with incident obesity between Phases 5 and 7 (Table 2).

### Antecedent Obesity Analysis

The results of linear regression analyses of the effect of prior obesity on Phase 5 sitting time are shown in Table 3. The group of participants classified as being obese at all three time points prior to Phase 5 watched an average of nearly 9 hours of TV per week more than the reference category (never obese at any measurement prior to Phase 5). Being obese on three occasions prior to Phase 5 was associated also with a 6-hour/week increase in total leisure-time sitting (Model A) relative to the reference category. These effects were only slightly attenuated in the fully adjusted Model B. Being obese at one measurement phase prior to Phase 5 was associated with around 2.5 hours/week higher TV-viewing time at Phase 5 but not total leisure-time sitting. There were no associations between prior obesity and work sitting, non-TV leisure-time sitting, or total sitting.

## Discussion

In analyses of data from a British occupational cohort study, no evidence of cross-sectional or prospective associations between five sitting time indicators and prevalent or incident obesity were found. Conversely, prior obesity was associated with higher levels of TV-viewing time at Phase 5. These findings are not consistent with several previous studies that have demonstrated positive prospective associations between sitting time and obesity,^14,19^ markers of body composition,^20^ and weight gain.^18^

One possible explanation for the lack of associations in the present data is a higher-than-average energy expenditure accrued as a consequence of walking and standing for transport in the Whitehall II cohort. The mean reported walking time for the whole sample was 40.71 (±20.83) minutes/day, which is considerably higher than the population average reported in the 2005 UK Time Use Survey (17 minutes/day).^34^ This difference may reflect the commuting habits of London professionals who, because of the public transport infrastructure, may be more likely to walk and stand (on buses and trains) on their journey to work, than people residing and working in other areas of the country who may be more accustomed to commuting by car.^35^ Hu et al.^14^ observed that although sitting time was positively associated with obesity risk, time spent standing or walking around was associated with a reduction in obesity risk.

In addition, it has previously been demonstrated that habitual active transport may moderate the association between TV viewing and obesity.^36,37^ The volume of MVPA reported by this cohort is also high in comparison with other prospective studies. It has been observed previously that London civil servants report higher levels of physical activity than the age-matched wider population.^38^ The total daily energy expenditure attributable to habitual active commuting and leisure-time physical activity is higher than that observed in other cohorts and may be sufficient to counter the risk of obesity due to prolonged sitting.

In the current analysis, obesity prior to Phase 5 was associated with TV viewing at Phase 5, although the association was not linear. The strongest association was in participants who were obese at all time points. These observations are consistent with findings from previous studies that have reported that measures of body weight and composition were prospectively associated with sitting time,^21^ having a sedentary lifestyle^22^ and reduced physical activity levels,^23,24^ while reporting no association in the other direction.

One such study^21^ observed that after adjustment for covariates, baseline sedentary time was not predictive of changes in body weight, BMI, fat mass, or waist circumference at follow-up. However, when the adiposity outcomes were modeled as exposure variables, all four independently predicted sitting time at follow-up. In the same study, changes in body weight, BMI, and fat mass between baseline and follow-up were predictive of sitting time at follow-up. Of the previous studies that have shown an association between indicators of sitting time and markers of obesity, only one adjusted for earlier BMI.^19^ A recent report of a UK birth cohort also found that following adjustment for baseline BMI, observed positive associations between TV-viewing frequency at age 23 years and cardiovascular risk factors and waist circumference at age 44 years were attenuated to null.^25^

The finding that an effect of prior obesity was associated only with time spent watching TV and leisure-time sitting is logical, as arguably people can exert more control over how much time they spend sitting at home compared to at work. TV viewing also may be easier to recall than other sitting behaviors, which may be more sporadic, and the greater recall error associated with these behaviors may attenuate any true association toward null. Sitting at work also may be less prone to recall error, but the present study has limited ability to detect associations between work sitting and obesity because of the lack of variance in work-related sitting among employees of the civil service.

The large sample size and prospective design are major strengths of the current study, as is the objective measurement of BMI by trained professionals. It also was possible to take account of a number of important confounding factors, notably employment grade, alcohol intake, self-rated health, physical activity, and physical functioning. Physical functioning could have an effect on sitting time, as physical limitation could dictate an individual's choice of leisure-time activity. Periods of limited physical functioning due to injury or ill health may somewhat artificially inflate an individual's reported sitting behavior and, if not considered, be a source of confounding. To the authors' knowledge, this is the first study to account for a measure of physical functioning when examining prospective associations between sitting time and obesity.

### Limitations

The present study is not without limitation. Occupational cohort participants are by definition sufficiently healthy to be in active employment, which may reduce the extent to which conclusions may be generalized to a wider population. Women are under-represented in this cohort, comprising approximately one quarter of the analysis groups. Individuals in the lowest employment grade also were under-represented in this sample, comprising only 11% in the cross-sectional analysis group, and only 9% in the prospective analysis group, with the remainder split approximately equally between the two higher employment grades. A recent prospective analysis of data from this cohort demonstrated that over a 10-year follow up period, individuals in higher employment grades showed smaller increases in waist circumference and BMI.^39^ Therefore, it is possible that the under-representation of the lower employment grades may have disproportionally reduced the incidence of obesity observed in the current sample.

The reliance on self-report measures may have led to misclassification of sitting, which, if nondifferential, would attenuate the association between sitting and obesity risk toward the null. A more precise measure of sitting time may have led to stronger associations. As BMI is a more precise measure than sitting, it also is possible that an association was more likely to be observed in the current study when obesity was modeled as an exposure. However, items used to construct the sitting variables in the current study have been used elsewhere,^14^ and validated.^29^ In addition, previous Whitehall II publications have shown associations between self-reported health behaviors, including physical activity, and obesity, suggesting that questionnaire items on health behavior have predictive validity.^40^

Previous studies have shown beneficial effects of leisure-time physical activity on obesity risk.^14^ Such effects are not evident in this cohort, which may in part be due to the omission of walking from the computation of this variable. Although analyses were adjusted for walking time, how much was of light or moderate intensity is unknown.

The results of the present study and others suggest a complex relationship in which the direction of the association between adiposity and sitting time is not entirely certain. Uncertainty also remains as to whether time spent sitting is simply a proxy for low total daily energy expenditure^41^ or whether sitting itself represents an independent risk for obesity. Further prospective or experimental research, with more precise measurement of time spent in specific sitting behaviors, is required to better determine if adiposity or weight gain leads to more sitting, or vice versa. Future studies also need a precise measurement of the potential confounding effect of energy balance.

### Conclusion

Time spent sitting while at work, TV viewing, and non-TV leisure-time sitting were not associated with incident or prevalent obesity in this occupational cohort. Prior obesity was associated with the amount of time an individual spent sitting while watching TV, suggesting that the relationship between sedentary behavior and obesity may be more complex than has been suggested previously. The possibility of reciprocal or reverse causality in this association requires further research attention.

## Figures and Tables

**Table 1:** Subject characteristics at baseline (Phase 5: 1997–1999)

	Sitting group (total from work and leisure time)			
	1 (*n*=408)	2 (*n*=562)	3 (*n*=496)	4 (*n*=505)
**Age (years)**	42.00 (5.00)	40.00 (4.00)	40.00 (4.00)	40.00 (4.00)
**Male (%)**	17.77	28.85	26.13	27.25
**Female (%)**	28.54	27.61	22.57	21.27
**BMI**	26.00 (4.10)	26.00 (3.80)	26.00 (3.80)	26.00 (4.10)
**Waist circumference (cm)**	88.20 (12.44)	88.19 (11.43)	88.78 (11.34)	89.57 (11.38)
**Weight (kg)**	77.00 (14.00)	77.00 (13.00)	78.00 (13.00)	79.00 (14.00)
**Walking (minutes/day)**	42.50 (20.96)	42.09 (20.12)	39.82 (20.01)	41.28 (20.28)
**MVPA (hours/week)**	10.86 (9.88)	12.10 (9.87)	12.42 (10.08)	11.74 (9.59)
**Employment grade (%)**				
Administrative	12.66	31.53	28.88	26.93
Prof/executive	23.33	26.95	23.78	25.93
Clerical/support	42.01	22.83	15.98	19.18
**Alcohol consumption (units/week)**	12.00 (14.00)	14.00 (14.00)	15.00 (15.00)	15.00 (16.00)
**Smoking status (%)**				
Never	19.98	28.68	25.62	25.72
Former	21.78	29.39	24.42	24.42
Current	20.87	24.76	25.24	29.13
**Self-rated health (%)**				
Very good	19.33	30.83	26.85	22.99
Good	20.93	26.32	24.94	27.82
Fair or poor	25.93	26.39	18.52	29.17

*Note:* Data are M (SD) unless otherwise stated. MVPA, moderate- to vigorous-intensity physical activity

**Table 2:** Obesity risk according to categories of sitting behaviors from cross-sectional and prospective analyses

Analysis	Sitting type	*n* (cases)	Ref OR	2 OR (95% CI)	3 OR (95% CI)	4 OR (95% CI)
Cross-sectional	Work	1954 (252)	1	1.21 (0.77, 1.88)	1.02 (0.68, 1.55)	1.03 (0.68, 1.55)
Phase 5 (1997–1999)	TV	1359 (183)		1.22 (0.70, 2.13)	1.35 (0.80, 2.28)	1.35 (0.77, 2.38)
	Non-TV leisure time	1200 (143)		1.05 (0.63, 1.74)	1.52 (0.93, 2.49)	0.80 (0.43, 1.46)
	Leisure time	1937 (251)		1.32 (0.91, 1.90)	0.94 (0.65, 1.37)	1.27 (0.83, 1.95)
	Total	1971 (256)		0.79 (0.53, 1.18)	0.89 (0.60, 1.34)	0.83 (0.56, 1.25)
Prospective	Work	1545 (97)	1	0.87 (0.43, 1.75)	0.85 (0.44, 1.62)	1.10 (0.59, 1.96)
Phases 5–7 (1997–2004)	TV	1071 (66)		0.99 (0.43, 2.24)	1.04 (0.48, 2.25)	0.97 (0.41, 2.29)
	Non-TV leisure time	959 (65)		1.07 (0.54, 2.11)	0.97 (0.48, 1.99)	0.88 (0.40, 1.95)
	Leisure time	1534 (96)		0.94 (0.53, 1.67)	1.03 (0.58, 1.83)	1.28 (0.67, 2.47)
	Total	1559 (98)		0.55 (0.30, 1.03)	0.79 (0.44, 1.43)	0.95 (0.51, 1.74)

*Note:* Adjusted for age, gender, employment grade, smoking status, weekly alcohol intake, self-rated health, physical functioning, daily walking time, and time spent in moderate- to vigorous-intensity physical activity. Analysis groups: For cross-sectional and prospective analyses: work, in hours per week: 1=0–20; 2=21–30; 3=31–39; 4= ≥40; TV-viewing time, in hours per week: 1=0–6; 2=7–11; 3=12–18; 4= ≥19; non-TV leisure time, in hours per week: 1=0–6; 2=7–11; 3=12–16; 4= ≥17. For cross-sectional analysis: leisure time, in hours per week: 1=0–11; 2=12–15; 3=16–25; 4= ≥25; Total, in hours per week: 1=0–33; 2=34–48; 3=49–56; 4= ≥57. For prospective analysis: leisure time, in hours per week: 1=0–9; 2=10–15; 3=16–25; 4= ≥26.

**Table 3:** Hours per week of sitting at Phase 5 according to occasions of prior obesity

	Sitting type									
	Work sitting (*n*=1858)		TV viewing (*n*=1286)		Non-TV leisure-time sitting (*n*=1146)		Leisure-time sitting (*n*=1843)		Total sitting (*n*=1874)	
Occasions of obesity	Model A	Model B	Model A	Model B	Model A	Model B	Model A	Model B	Model A	Model B
0	0	0	0	0	0	0	0	0	0	0
1	−1.96 (−4.55, 0.61)	−2.13 (−4.66, 0.38)	**2.72 (0.36, 5.08)**	**2.43 (0.07, 4.78)**	−1.81 (−4.68, 1.06)	−2.07 (−4.98, 0.83)	0.39 (−1.98, 2.77)	0.06 (−2.32, 2.44)	−1.89 (−5.67, 1.87)	−2.45 (−6.21, 1.31)
2	−0.18 (−3.18, 2.81)	0.70 (−2.22, 3.63)	−0.33 (−2.71, 2.64)	−0.62 (−3.29, 2.04)	−0.82 (−4.37, 2.72)	−1.13 (−4.74, 2.47)	−0.92 (−3.72, 1.88)	−1.46 (−4.27, 1.34)	−0.87 (−5.30, 3.56)	−0.74 (−5.16, 3.68)
3	0.99 (−4.82, 6.82)	2.57 (−3.11, 8.25)	**8.78 (3.73, 13.84)**	**7.41 (2.36, 12.46)**	−2.76 (−9.35, 3.82)	−2.87 (−9.49, 3.76)	**5.91 (0.51, 11.31)**	**5.20 (0.19, 10.60)**	7.47 (−1.14, 16.08)	8.24 (−0.33, 16.82)

*Note:* Boldface indicates significance. Obesity was classified from recalled weight at age 25 years (from Phase-1 questionnaire), and BMI at Phases 1 and 3. Coefficients are sitting time (hours/week). Model A is adjusted for age and gender only. Model B is additionally adjusted for employment grade, smoking status, weekly alcohol intake, self-rated health, physical functioning, daily walking time, and time spent in moderate- to vigorous-intensity physical activity.

## Box

See related Commentary by Marshall in this issue.

## References

[1] Klein S., Allison D.B., Heymsfield S.B. (2007). Waist circumference and cardiometabolic risk: a consensus statement from Shaping America's Health: Association for Weight Management and Obesity Prevention; NAASO, the Obesity Society; the American Society for Nutrition; and the American Diabetes Association. Obesity (Silver Spring).

[2] Garber C.E., Blissmer B., Deschenes M.R., American College of Sports Medicine position stand (2011). Quantity and quality of exercise for developing and maintaining cardiorespiratory, musculoskeletal, and neuromotor fitness in apparently healthy adults: guidance for prescribing exercise. Med Sci Sports Exerc.

[3] Warburton D.E., Nicol C.W., Bredin S.S. (2006). Health benefits of physical activity: the evidence. CMAJ.

[4] Healy G.N., Wijndaele K., Dunstan D.W. (2008). Objectively measured sedentary time, physical activity, and metabolic risk: the Australian Diabetes, Obesity and Lifestyle Study (AusDiab). Diabetes Care.

[5] Stamatakis E., Hamer M. (2011). Sedentary behaviour: redefining its meaning and links to chronic disease. Br J Hosp Med (Lond).

[6] Dunstan D.W., Barr E.L., Healy G.N. (2010). Television viewing time and mortality: the Australian Diabetes, Obesity and Lifestyle Study (AusDiab). Circulation.

[7] Katzmarzyk P.T., Church T.S., Craig C.L., Bouchard C. (2009). Sitting time and mortality from all causes, cardiovascular disease, and cancer. Med Sci Sports Exerc.

[8] Patel A.V., Bernstein L., Deka A. (2010). Leisure time spent sitting in relation to total mortality in a prospective cohort of U.S. adults. Am J Epidemiol.

[9] Warren T.Y., Barry V., Hooker S.P., Sui X.M., Church T.S., Blair S.N. (2010). Sedentary behaviors increase risk of cardiovascular disease mortality in men. Med Sci Sports Exerc.

[10] Stamatakis E., Hamer M., Dunstan D.W. (2011). Screen-based entertainment time, all-cause mortality, and cardiovascular events: population-based study with ongoing mortality and hospital events follow-up. J Am Coll Cardiol.

[11] Wijndaele K., Brage S., Besson H. (2011). Television viewing and incident cardiovascular disease: prospective associations and mediation analysis in the EPIC Norfolk Study. PLoS One.

[12] Ford E.S., Schulze M.B., Kroger J., Pischon T., Bergmann M.M., Boeing H. (2010). Television watching and incident diabetes: findings from the European Prospective Investigation into Cancer and Nutrition–Potsdam Study. J Diabetes.

[13] Hu F.B., Leitzmann M.F., Stampfer M.J., Colditz G.A., Willett W.C., Rimm E.B. (2001). Physical activity and television watching in relation to risk for type 2 diabetes mellitus in men. Arch Intern Med.

[14] Hu F.B., Li T.Y., Colditz G.A., Willett W.C., Manson J.E. (2003). Television watching and other sedentary behaviors in relation to risk of obesity and type 2 diabetes mellitus in women. JAMA.

[15] Krishnan S., Rosenberg L., Palmer J.R. (2009). Physical activity and television watching in relation to risk of type 2 diabetes: the Black Women's Health Study. Am J Epidemiol.

[16] Jakes R.W., Day N.E., Khaw K.T. (2003). Television viewing and low participation in vigorous recreation are independently associated with obesity and markers of cardiovascular disease risk: EPIC-Norfolk population-based study. Eur J Clin Nutr.

[17] Stamatakis E., Hirani V., Rennie K. (2009). Moderate-to-vigorous physical activity and sedentary behaviours in relation to body mass index-defined and waist circumference-defined obesity. Br J Nutr.

[18] Blanck H.M., McCullough M.L., Patel A.V. (2007). Sedentary behavior, recreational physical activity, and 7-year weight gain among postmenopausal U.S. women. Obesity (Silver Spring).

[19] Parsons T.J., Manor O., Power C. (2008). Television viewing and obesity: a prospective study in the 1958 British birth cohort. Eur J Clin Nutr.

[20] Wijndaele K., Healy G.N., Dunstan D.W. (2010). Increased cardiometabolic risk is associated with increased TV viewing time. Med Sci Sports Exerc.

[21] Ekelund U., Brage S., Besson H., Sharp S., Wareham N.J. (2008). Time spent being sedentary and weight gain in healthy adults: reverse or bidirectional causality?. Am J Clin Nutr.

[22] Mortensen L.H., Siegler I.C., Barefoot J.C., Gronbaek M., Sorensen T.I. (2006). Prospective associations between sedentary lifestyle and BMI in midlife. Obesity (Silver Spring).

[23] Lakerveld J., Dunstan D., Bot S. (2011). Abdominal obesity, TV-viewing time and prospective declines in physical activity. Prev Med.

[24] Golubic R., Ekelund U., Wijndaele K. (2012). Rate of weight gain predicts change in physical activity levels: a longitudinal analysis of the EPIC-Norfolk cohort. Int J Obes.

[25] Stamatakis E., Hamer M., Mishra G.D. (2012). Early adulthood television viewing and cardiometabolic risk profiles in early middle age: results from a population, prospective cohort study. Diabetologia.

[26] Stamatakis E., Hamer M. (2012). The extent to which adiposity markers explain the association between sedentary behavior and cardiometabolic risk factors. Obesity (Silver Spring).

[27] Healy G.N., Dunstan D.W., Salmon J. (2008). Breaks in sedentary time—beneficial associations with metabolic risk. Diabetes Care.

[28] Marmot M., Brunner E. (2005). Cohort profile: the Whitehall II study. Int J Epidemiol.

[29] Wolf A.M., Hunter D.J., Colditz G.A. (1994). Reproducibility and validity of a self-administered physical activity questionnaire. Int J Epidemiol.

[30] Marmot M.G., Smith G.D., Stansfeld S. (1991). Health inequalities among British civil servants: the Whitehall II study. Lancet.

[31] Ware J.E., Snow K.K., Kosinski M. (1993). SF-36 health survey manual and interpretation guide.

[32] McHorney C.A., Ware J.E., Raczek A.E. (1993). The MOS 36-Item Short-Form Health Survey (SF-36): II: Psychometric and clinical tests of validity in measuring physical and mental health constructs. Med Care.

[33] Ainsworth B.E., Haskell W.L., Whitt M.C. (2000). Compendium of physical activities: an update of activity codes and MET intensities. Med Sci Sports Exerc.

[34] Office for National Statistics (2006). The Time Use Survey 2005.

[35] Department for Transport (2011). National Travel Survey 2010.

[36] Sugiyama T., Merom D., Reeves M., Leslie E., Owen N. (2010). Habitual active transport moderates the association of TV viewing time with body mass index. J Phys Act Health.

[37] Ding D., Sugiyama T., Owen N. (2012). Habitual active transport, TV viewing and weight gain: a four year follow-up study. Prev Med.

[38] Morris J.N., Clayton D.G., Everitt M.G., Semmence A.M., Burgess E.H. (1990). Exercise in leisure time: coronary attack and death rates. Br Heart J.

[39] Elovainio M., Ferrie J.E., Singh-Manoux A. (2011). Socioeconomic differences in cardiometabolic factors: social causation or health-related selection?: Evidence from the Whitehall II Cohort Study, 1991–2004. Am J Epidemiol.

[40] Brunner E.J., Chandola T., Marmot M.G. (2007). Prospective effect of job strain on general and central obesity in the Whitehall II Study. Am J Epidemiol.

[41] Stephens B.R., Granados K., Zderic T.W., Hamilton M.T., Braun B. (2011). Effects of 1 day of inactivity on insulin action in healthy men and women: interaction with energy intake. Metabolism.

